# The 3′-Terminal Hexamer Sequence of *Classical swine fever virus* RNA Plays a Role in Negatively Regulating the IRES-Mediated Translation

**DOI:** 10.1371/journal.pone.0033764

**Published:** 2012-03-14

**Authors:** Shih-Wei Huang, Meng-Yu Chan, Wei-Li Hsu, Chin-Cheng Huang, Ching-Hsiu Tsai

**Affiliations:** 1 Graduate Institute of Biotechnology, National Chung Hsing University, Taichung, Taiwan; 2 Graduate Institute of Microbiology and Public Health, National Chung Hsing University, Taichung, Taiwan; 3 Department of Hog Cholera, Animal Health Research Institute, Council of Agriculture, Taipei, Taiwan; 4 Graduate Institute of Medical Laboratory Science and Biotechnology, China Medical University, Taichung, Taiwan; Pohang University of Science and Technology, Republic of Korea

## Abstract

The 3′ untranslated region (UTR) is usually involved in the switch of the translation and replication for a positive-sense RNA virus. To understand the 3′ UTR involved in an internal ribosome entry site (IRES)-mediated translation in *Classical swine fever virus* (CSFV), we first confirmed the predicted secondary structure (designated as SLI, SLII, SLIII, and SLIV) by enzymatic probing. Using a reporter assay in which the luciferase expression is under the control of CSFV 5′ and 3′ UTRs, we found that the 3′ UTR harbors the positive and negative regulatory elements for translational control. Unlike other stem loops, SLI acts as a repressor for expression of the reporter gene. The negative *cis*-acting element in SLI is further mapped to the very 3′-end hexamer CGGCCC sequence. Further, the CSFV IRES-mediated translation can be enhanced by the heterologous 3′-ends such as the poly(A) or the 3′ UTR of *Hepatitis C virus* (HCV). Interestingly, such an enhancement was repressed by flanking this hexamer to the end of poly(A) or HCV 3′ UTR. After sequence comparison and alignment, we have found that this hexamer sequence could hypothetically base pair with the sequence in the IRES IIId1, the 40 S ribosomal subunit binding site for the translational initiation, located at the 5′ UTR. In conclusion, we have found that the 3′-end terminal sequence can play a role in regulating the translation of CSFV.

## Introduction

*Classical swine fever virus* (CSFV), a member of the genus *Pestivirus* of the family *Flaviviridae* [1], is the causative agent of classical swine fever (CSF), previously known as hog cholera. CSF is highly contagious and often causes fatal hemorrhagic disease in pigs, resulting in highly significant economic losses worldwide [2], [3]. The genome of CSFV is a single-stranded, positive-sense RNA of approximately 12.3 kb in length, neither capped at its 5′-end nor polyadenylated at its 3′-end. The genomic RNA comprises a single open reading frame (ORF), flanked by the 5′ and the 3′ untranslated regions (UTRs), which serves as mRNA for the synthesis of a single large polyprotein of 3,898 amino acids [4]. The polyprotein precursor is further processed into mature structural and nonstructural proteins by cleavage of viral and cellular proteases. From the N- to the C-terminus, the viral proteins are arranged in the following order: Npro, C, Erns, E1, E2, P7, NS2, NS3, NS4A, NS4B, NS5A and NS5B [5].

Viral protein synthesis of CSFV is initiated in a cap-independent manner by a highly conserved structure known as internal ribosome entry site (IRES), located in the 5′ UTR. The structure and function of CSFV IRES is similar to that of the well-characterized *Hepatitis C virus* (HCV), comprising two main structural domains marked as II and III [6]. The IRES of both viruses can capture the 40 S ribosomal subunit directly without any known initiation factor [7]. The domain III of HCV IRES is the major anchoring site for the 40 S ribosomal subunit and recruits translation initiation factor eIF3 for efficient 80 S complex formation [8]. The domain II is essential for the conformational change of 40 S that allows the mRNA to go into the ribosomal binding channel [9], and promotes GTP hydrolysis to release eIF2 [10].

A number of cellular RNA-binding proteins have been identified as IRES-transacting factors (ITAFs), which play various roles in a number of biological situations [11], [12]. These ITAFs such as polypyrimidine tract-binding proteins [13], poly(C)-binding protein [14], NS1-associated protein1, and La auto-antigen [15] could bind to the viral IRES and modulate the gene expression as non-canonical translation factors. For the IRES-containing viral RNA genome, the 3′ UTR is usually in a highly ordered structure and harbors the signals for replication and translation in the viral life cycle [16]. During translation, the viral RNAs as well as cellular messenger RNAs can form a “closed-loop” structure through the 5′-3′ RNA-RNA or RNA-protein interaction [17]. This concept of end-to-end communication has been reported to contribute to viral translation stimulation [17], [18], [19], replication [20], [21], [22], and the switch between protein synthesis and RNA replication [23]. However, translational control by which the 3′ UTR or its binding proteins regulate IRES-mediated translation has not been discussed extensively.

Here we investigate the 3′ UTR of CSFV that participates in its translation regulation. First, we determined the secondary structure of the 3′ UTR of CSFV by enzymatic probing. Second, we constructed the reporter system by transfecting the RNA into cells with the UTRs of CSFV flanking the luciferase gene. We found that the 3′ UTR of CSFV could positively and negatively regulate the IRES-dependent translation. Furthermore, our results also suggest that the hexamer sequence of the 3′-terminus CGGCCC can shut down the translation, possibly by a long distance RNA-RNA interaction with the apical loop of domain IIId1 in IRES.

## Materials and Methods

### Plasmid constructs

The cDNA clones of CSFV containing the 3′ UTRs of ALD or LPC strain [3] were used as the templates for polymerase chain reaction (PCR) to generate the fragments containing a T7 RNA promoter for structural probing. The PCR primers used in the reaction are: CSFV/5′T7+12,058 (5′TAATACGACTCACTATAGGGTATGAGCGCGGGTAACCCGGGATCTGGA3′), ALD3′12,328 (5′GGGCCGTTAGGAAATTACCTTAGTC3′) and LPC3′12,269 (5′GGGCCGTTAGAAATTACCTTA3′). The T7 promoter sequence is underlined. The PCR products were cloned into pUC18 and verified by sequencing.

To generate the LPC3′SLIII (ALD3′SLIII), LPC3′SLII/I, and LPC3′SLI transcripts, DNA fragments were amplified from the LPC 3′ UTR clone by PCR and transcribed directly. LPC3′SLIII and ALD3′SLIII were generated using LPCT7+SLIII/5′ (5′TAATACGACTCACTATAGGACCCTATTGTAGA3′) and LPCSLIII/3′ (5′CAATAAATAAATAAATAAATAAA3′); LPC3′SLII/I using LPCT7+12,182/5′ (5′TAATACGACTCACTATAGGGAATGAGTAAGAATT 3′) and LPC-12,269/3′ (5′GGGCCGTTAGAAATTACCTTA3′); and LPC3′SLI using LPCT7+12,252/5′ (5′TAATACGACTCACTATAGGGCTGGAAGGAAAA3′) and LPC3′12,269.

Constructs for translation studies were all derived from plasmid pGEM3Z/ALD containing the full-length cDNA of CSFV. At first, pGEM3Z/ALD was digested by *Kpn* I and then self-ligated to remove the 7-kb fragment of CSFV coding region. The resulting clone was then digested with *Apa*I, and followed by a self-ligation to obtain the clone pALD/ΔAK/A that contains UTRs. For the *in vivo* translation assay, pALD/L/A were derived from pALD/ΔAK/A. At first, p2Luc containing the firefly luciferase was used as the template for PCR to generate the fragments containing *Apa* I and *Spe* I with the 5′ primer Apa L (5′GAA*GGGCCC*CGAAGACGCCAAAAACATAAAGAA3′) and the 3′primer Lspe (5′GAA*ACTAGT*TTACAATTTGGACTTTCCGCCCTT3′). The restriction enzyme site is in Italic. The relative DNA fragment in pALD/ΔAK/A was replaced with the PCR-amplified fragments containing luciferase gene by digestion with *Apa* I and *Spe* I. The resulting plasmid pALD/L/A containing a T7 promoter, the ALD 5′ UTR (nucleotides 1 to 374, numbering is from CSFV genomic RNA), partial Npro protein coding sequence, firefly luciferase gene (L), and ALD 3′ UTR. The replacement of the 3′ UTR of CSFV with poly (A) tail (30A) or the 3′ UTR of HCV was done by PCR with *Spe* I containing forward and *Hin*d III containing reverse primers (Table 1). The PCR products were cloned into T-easy vector (Promega, Madison, WI, USA). The sequences were verified and sub-cloned into pALD/L/A with *Spe* I and *Hin*d III sites. The resulting plasmids were designated as pALD/L/(A)_n_ and pALD/L/H, respectively. The rest of constructs, pALD/L/SLI, -/SLII, -/SLIII, -/AupSLI, and -/AlowSLI, with the mutations in the 3′ UTR of pALD/L/A were constructed using the same approach. To generate the plasmids pALD/L/AupSLI and pALD/L/AlowSLI, the 3′ reverse primers were the megaprimers obtained by first PCR with the primers (primer 1 and primer 2) indicated in the Table 1.

**Table 1:** Primers used to construct the clones for in vitro transcription.

Plasmid clone	5′ forward primer	3′ reverse primer	template
pTEasy-AS-Luc	GAAGGGCCCCGAAGACGCCAAAAACATAAAGAA	GAAACTAGTTTACAATTTGGACTTTCCGCCCTT	p2Luc
pALD/L/A	GACTAGTGGGTATGAGCGCGG GTAACCC	GAAGCTTGCATG CCTGCAGG CCC	pALD/ΔAK/A
pALD/L/(A)_n_	GACTAGTA_29_	GAAGCT_32_	-
pALD/L/H	GACTAGTACGGGGAGCTAAACACTCCA	GAAGCTTACTTGAT CTGCAGAGAGGCC AGTATC	HCV replicon
pALD/L/SLI	GACTAGTCACTTTAGCTGGAA GGAAAA	GAAGCTTGCATGCC TGCAGG CCC	pALD/ΔAK/A
pALD/L/SLII	GACTAGTTTTATTGAATGAGT AAGAAC	GAAGCTTCTGTTAA AAATGAG TGTAGT	pALD/ΔAK/A
pALD/L/SLIII	GACTAGTTTGTAGATAACACTAATTTT	GAAGCTTTAAATAA ATAAATAA ATAGT	pALD/ΔAK/A
pALD/L/AupSLI	GACTAGTGGGTATGAGCGCGG GTAACCC	a ACTCATTTTTAACAGCCTGACGTCCACAGT (primer 1); GAAGCTTCCTTAGTCCAACTGTGGACGTCAG GCTGTT (primer 2)	pALD/ΔAK/A
pALD/L/AlowSLI	GACTAGTGGGTATGAGCGCGG GTAACCC	a ACTCATTTTTAACAGCACTTTAGCTGGAAGGAAAATTTAA (primer 1); GAAGCTTGGGCCGTTAGGAAATTAAATTTTCCTTCCAGCTA (primer 2)	pALD/ΔAK/A

a The two primers (indicated as primer 1 and primer 2) were used for first PCR to synthesize the megaprimer as the 3′ reverse primer for the second PCR.

### In vitro transcription

The templates used for *in vitro* transcription were mostly derived from PCR fragments except ALD/L/(A)_n_ which was cleaved with *Hin*d III for run-off transcription. The gel-purified PCR products for transcription were generated using the common 5′ primer (5′GACGTCTAAGAAACCATTATTATC3′) and the specific 3′ primer (Table 2).

**Table 2:** Primers used to generate the PCR fragments directly for in vitro transcription.

RNA	Plasmid templates	3′ primer sequence
ALD/L/Δ	pALD/L/A	TTACAATTTGGACTTTCCGC
ALD/L/A	pALD/L/A	GGGCCGTTAGGAAATTACCTTA
ALD/L/H	pALD/L/H	ACTTGATCTGCAGAGAGGCCAGTATC
ALD/L/AΔSLI	pALD/L/A	CTGTTAAAAATGAGTGTAGTGTGGT
ALD/L/AΔSLISLII	pALD/L/A	TAAATAAATAAATAAATAGTAATAT
ALD/L/SLIV	pALD/L/A	TAGGGTCCTACTGGCGGGTCCAGAT
ALD/L/SLIII	pALD/L/SLIII	TAAATAAATAAATAAATAGTAATAT
ALD/L/SLII	pALD/L/SLII	CTGTTAAAAATGAGTGTAGTGTGGT
ALD/L/SLI	pALD/L/SLI	GGGCCGTTAGGAAATTACCTTA
ALD/L/AupSLI	pALD/L/AupSLI	CCTTAGTCCAACTGTGGACGTCAGGC
ALD/L/AlowSLI	pALD/L/AlowSLI	GGGCCGTTAGGAAATTAAATTTTCCTTC
ALD/L/AlowSLI-L	pALD/L/AlowSLI	AATTTTCCTT CCAGCTAAAG
ALD/L/AlowSLI-R	pALD/L/AlowSLI	GGGCCGTTAGGAAATTACTGTTAAAAATGA GTGTAGT
ALD/L/AΔSLI-UCCUAA	pALD/L/A	TTAGGACTGTTAAAAATGAGTGTAGT
ALD/L/AΔSLI-CGGCCC	pALD/L/A	GGGCCGCTGTTAAAAATGAGTGTAGT
ALD/L/(A)n-CGGCCC	ALD/L/(A)n	GGGCCGT_27_
ALD/L/(A)n-UCCUAACGGCCC	ALD/L/(A)n	GGGCCGTTAGGAT_27_
ALD/L/H-CGGCCC	ALD/L/H	GGGCCGACTTGATCTGCAGAGAGGCCA
ALD/L/H- UCCUAACGGCCC	ALD/L/H	GGGCCGTTAGGAACTTGATCTGCAGAGAGGCCAGTATC

*In vitro* transcription was carried out at 37°C for 2 h in a 50 µl reaction containing 150 U of T7 RNA polymerase, 40 mM Tris-HCl pH 8.0, 8 mM MgCl_2_, 2 mM spermidine-(HCl)_3_, 10 mM dithiothreitol, 1 mM ATP/UTP/CTP/GTP. DNA templates was removed by digestion with RNase-free DNase I, and followed by a phenol/chloroform extraction, ethanol precipitation, and salt precipitation. RNAs were resolved through a 1% agarose gel to analyze the RNA quality and quantify the yield by a densitometry.

### RNA labeling

To label the 5′-ends of the LPC3′UTR, LPC3′SLII/I, and LPC3′SLI transcripts, 1.5 µg of gel-purified transcripts were dephosphorylated first and then treated with T4 polynucleotides kinase [24]. Labeled transcripts were purified by electrophoresis on a 10% sequencing gel and eluted.

### Structure prediction and mapping of the CSFV 3′UTR

As predicted by mfold [25], 3′ UTR of ALD and LPC strains form four stem-loops. Labeled LPC3′UTR, LPCSLII/I, and LPCSLI were cleaved with specific RNases. Labeled RNAs were partially digested with the alkaline buffer (55.5 mM Na/carbonate pH 9.0 and 1.1 mM EDTA) and used as markers. Besides, the labeled RNAs were digested with RNase T1 (10 to 15 units) at 55°C or RNase A (3 ng) on ice for 10 minutes, and subsequently denatured in boiling water for 90 and 30 sec, respectively, to serve as the sequencing ladder markers.

Digestion with RNase A, T1, T2, or V1 was performed at the condition described [26]. Serial dilutions of RNases were added, including 36 ng (100×) to 12 ng (300×) of RNase A, 5 to 0.25 units of RNase T1, 6 to 2 units of RNase T2 and 0.1 to 0.025 units of RNase V1.

### Cell culture, transfection and analysis of luciferase activity

Porcine kidney-15 (PK-15; ATCC CCL-33) cells were maintained in Dulbecco's modified Eagle medium (DMEM) supplemented with 10% fetal bovine serum (FBS), 1% penicillin-streptomycin at 37°C, and supplying with 5% CO_2_. About 4×10^5^ cells were seeded into 12-well plates and grown to 90% confluence. The 4 µl of Lipofectamine (Invitrogen, Carlsbad, CA, USA) and 0.8 µg of the transcribed RNA were each diluted with 100 µl of Opti-MEM and incubated at room temperature for 5 min. Both of the diluted components were mixed and incubated at room temperature for 25 min, and then added to the culture. For the harvest, cells were washed with phosphate-buffered saline (PBS), and about 150 µl of passive lysis buffer (Promega) was added to each well, and the cells were scraped, collected, and with a freeze-thaw process. After a centrifugation at 13,000×*g* for 5 min, 20 µl of lysate was mixed with 100 µl of Luciferase Assay reagent, and the firefly luciferase activity was measured by a luminometer (FLUOstar OPTIMA, BMG Labtech, Ortenberg, Germany).

## Results

### Structural prediction and enzymatic probing of the 3′ UTR of CSFV

**Figure 1:**
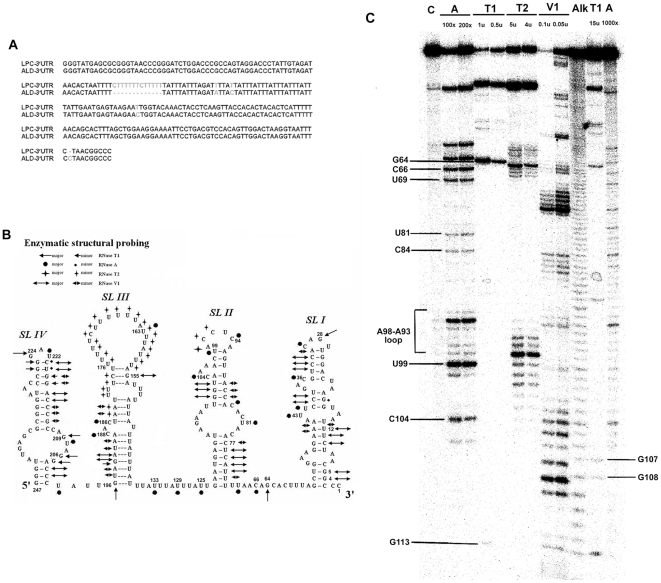
RNA sequence alignment and the enzymatic probing of the 3′ UTRs of *Classical swine fever virus*. **A.** Sequence alignment of the ALD3′UTR and LPC3′UTR. “−” represents the missing nucleotide. **B.** Schematic summary of the enzymatic structural probing results. The stem loops are designated as SLI (nt3 to nt56), SLII (nt71 to nt123), SLIII (nt137 to nt196), and SLIV (nt201 to nt247). Nucleotides are numbered from the 3′-end. Positions of the cleavages by single- or double-stranded RNA-specific probes are indicated by symbols as indicated in the figure. The AU-rich stem in SLIII was shown as dash line stands for unstable structure sensitive to both single- and double-stranded specific RNases. **C.** Enzymatic probing assays of the 5′ end-labeled LPCSLII/I. The RNAs were treated with RNase A (lane A), RNase T1 (lane T1), RNase T2 (lane T2), or RNase V1 (lane V1). The concentration of the enzymes used in each reaction is indicated above each lane. The control reaction (lane C) included LPC3′UTR without the addition of any RNase, and lane Alk was partial digestion of LPC 3′UTR with an alkaline buffer to serve as markers. Lane T1 (15 U) and lane A (1000×) were included as RNA sequencing markers. The resulted RNA fragments were resolved on a 10% sequencing gel.

The 3′ UTR sequences of CSFV strains ALD and LPC [3] were aligned using the CLUSTAL W program [27]. The major difference between these two strains is the presence of a thirteen-nucleotide sequence (CUUUUUUCUUUUU) in the 3′ UTR of LPC (Fig. 1A), which is similar to other lapinized vaccine strains such as C- and HCLV strain [3], [28], [29], [30]. The 3′ UTRs of these two strains, predicted with the mfold program [25], appeared to fold similarly into four consecutive independent stem loops, designated as SLI, SLII, SLIII, and SLIV from the 3′ end (Fig. 1B), in agreement with previous predictions [31]. The extra thirteen-nucleotide region of LPC was found to lie in the loop structure of SLIII.

To confirm the predicted structure of the 3′ UTR in solution, four RNases were used to probe the structure of the 3′ UTR of CSFV. A summary of the enzymatic structural probing results is shown in figure 1B. A representative result of the probing experiments is shown in figure 1C. Due to the difficulties encountered using the 5′ end-labeled LPC3′UTR transcripts to clearly and completely map the structures of SLII and SLI, shorter transcripts LPCSLII/I (Fig. 1C) and LPCSLI (Fig. S1) were generated.

Using LPC3′UTR as the substrate, appearance of RNase A-specific bands corresponding to the C104 (bulge), C94 (apical loop), C84, and U81 (internal loop) positions strongly substantiate their predicted locations within single-stranded regions. The production of a strong signal at G64 by RNase T1 also suggests that G64 is located in a single-stranded region, in agreement with the prediction. Efficient cleavage of nucleotides U109 to A105, A88 to A85, and A78 to U71 by RNase V1 digestion, as indicated by the prominent cleavage product bands, strongly supports the premise that they are located in the predicted stem regions. RNase T2 digestion resulted in the production of the products at A98 to A93 and U70 to A57, corresponding to the predicted apical loop region, and the single-stranded junction between SLII and SLI, respectively. Overall, the results of RNases T2 and V1 digestion, specific for single-strand RNA and double-strand RNA, respectively, did not overlap with each other (Fig. 1C). Similar cleavage patterns with the single-strand specific RNases A, T1, and T2, and with the double-strand specific RNase V1 on SLI, SLII, SLIII, and SLIV were matched to that of the predicted structure (Fig. S1, S2, and S3). Overall, these enzymatic structural probing results correlate considerably well with the existence of four independent stem-loops in the 3′ UTR of CSFV as predicted by computer analysis.

### The 3′ UTR of CSFV can stimulate its IRES-dependent translation in cells

**Figure 2:**
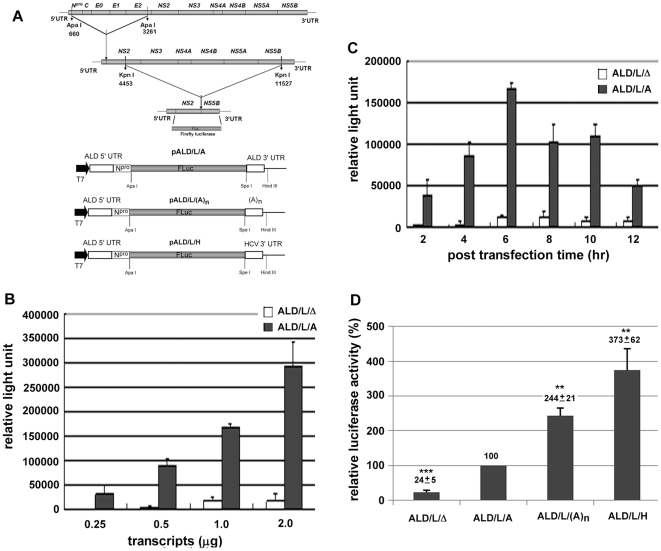
The 3′ UTR of CSFV is involved in IRES-mediated translation in PK-15 cells. **A.** Schematic diagram of CSFV reporter constructs. T7: T7 promoter; Npro: partial sequences of N protease; Fluc: firefly luciferase. **B.** Firefly luciferase activity was measured 4 hours of post-transfection with indicated amount of ALD/L/Δ or ALD/L/A RNA in PK-15 cells. **C.** Firefly luciferase activity was measured at different post transfection time points indicated with 1 pmole RNA in PK-15 cells. **D.** IRES-mediated translation stimulated by CSFV 3′ UTR, poly A tail and HCV 3′ UTR. All the transfection experiments have been independently repeated at least three times. The relative luciferase activity with standard deviations and their significances (*t*-test) was shown above each statistic bar. Asterisks indicate statistically significant differences compared with the wild type construct indicated as ALD/L/A (**p<0.01, ***p<0.001).

Previously, we tested the effect of the 3′ UTR derived from ALD and LPC strains, and found that the structural difference of the 3′ UTR could be involved in translation efficiency in an *in vitro* translation system (unpublished data). Therefore, we systematically evaluated the roles of the 3′ UTR participating in the regulation of translation in a cell-based system. The first, the reporter plasmid pALD/L/A, was generated by replacing nearly the entire coding region of CSFV with firefly luciferase gene (Fig. 2A). This plasmid turned out to contain a T7 promoter, 5′ UTR of ALD, N-terminal region of N^pro^ fused to the firefly luciferase gene, and the 3′ UTR of ALD (Fig. 2A). We then optimized the conditions for the transfection by evaluating the translation efficiency. However, we have observed that the untranslated regions could cause the cap-dependent translation shutdown (unpublished data). Therefore, the capped reporter control used for normalization [32], [33] showed varied expression when co-transfected with the RNAs containing different structure of the CSFV 3′ UTR. To avoid the varied expression caused by cap-dependent translation shutdown, most of the transfection experiments with the cap-independent CSFV RNAs were repeated at least three times. The translation efficiency in PK-15 cells is in a 3′ UTR- and dose-dependent manner (Fig. 2B). The translation efficiency, measured as the activity of firefly luciferase, reached maximum at six hours after transfection (Fig. 2C). Results indicate that the translation of the RNA containing the 3′ UTR of ALD was about 10 to 54-fold higher than that of the RNA without 3′ UTR (ALD/L/Δ) (Fig. 2B), indicating that the 3′ UTR of CSFV can strongly stimulate its IRES-dependent translation in cells.

### The poly(A) tail and the 3′ UTR of HCV enhance the CSFV IRES-mediated translation

Although the IRESes in the 5′ UTR of the related viruses from the *Flaviviridae* are structurally conserved, the 3′ UTRs of these viruses are distinctive from each other. The 3′ UTR of HCV was shown to facilitate the assembly of the translation initiation complex and enhance the IRES-mediated translation activity [34], [35], [36]. Since the IRES of HCV is structurally similar to that of CSFV, we tested whether the 3′ UTR of HCV can also regulate the IRES of CSFV in translation. A chimera RNA containing the 5′ UTR of CSFV but with the 3′ UTR of HCV was generated (Fig. 2A). Poly(A) sequence has been reported to have a general stimulatory effect not only on cap-dependent translation but also on cap-independent translation [34], [36], [37]. We then generated the RNA with the poly(A) tail to replace the 3′ UTR of CSFV, to test the translation (Fig. 2A). Results indicate that the firefly luciferase activity of the reporter system with poly(A) tail and the 3′ UTR of HCV increased about 2.5 and 3.8-fold of that with the 3′ UTR of CSFV, respectively (Fig. 2D).

### SLI in the 3′ UTR of CSFV has a negative effect on translation

**Figure 3:**
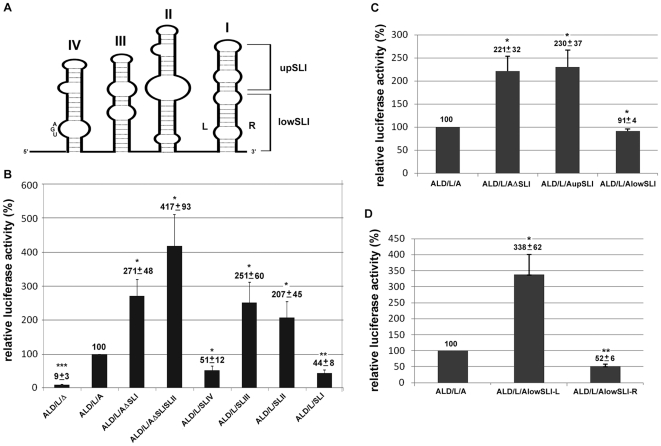
The characterization of stem-loop 1 in the 3′ UTR involved in the CSFV IRES-mediated translation. **A.** The diagram of the secondary structure of CSFV 3′ UTR. **B.** **C.** **D.** Firefly luciferase activity of the reporter transcripts (1 pmole) containing the wild type 3′ UTR (ALD/L/A) and its derivatives at 6 hours of post tranfection in PK-15 cells. All the transfection experiments have been independently repeated at least three times. The relative luciferase activity with standard deviations and their significances (*t*-test) was shown above each statistic bar. Asterisks indicate statistically significant differences compared with the control construct indicated as ALD/L/A (*p<0.05, **p<0.01, ***p<0.001).

We wondered why the 3′ UTR of CSFV was 2 to 4-fold less efficient in assisting its own IRES-mediated translation than those of heterologous 3′ UTR or poly(A) tail. Since the 3′ UTR of CSFV is composed of four stem-loops (Fig. 1 and 3A), we tested whether these stem-loops (SLs) may have a negative role in regulating translation. We generated a series of deletion mutants to determine the contribution of these four SLs in the 3′ UTR of CSFV in translation (Fig. 3B). The translation activity increased 2.7- or 4.1-fold when SLI alone (ALD/L/AΔSLI) or SLI and SLII (ALD/L/AΔSLISLII) is deleted, respectively. The resulting activity of these mutants similar to that of the constructs with the poly(A) tail or the 3′ UTR of HCV (Fig. 2D). These results imply that each of the stem-loops in the 3′ UTR of CSFV may have different roles in regulating the translation. A series of reporter constructs each with one of the individual stem-loops of 3′ UTR was used to evaluate their contribution to translation activity. Results indicate that both the SLII and SLIII had a positive effect, but SLI and SLIV had a negative effect on the translation compared to that of the entire 3′ UTR (Fig. 3B). Considering that luciferase activity increased in the absence of SL1,i.e. ALD/L/AΔSLI (2.7-fold increase), and ALD/L/AΔSLISLII (4.1-fold increase), but in the presence of SL1 (ALD/L/SLI), it reduced to less than 50% of that with entire 3′ UTR, suggests that SLI has a negative regulation in CSFV IRES-mediated translation. Although SLI or SLIV in the 3′ UTR of CSFV plays a negative role in translation, they somehow contribute to the translation about 5 to 6 folds compared to that of ALD/L/Δ (Fig. 3B).

To pin down the region of SLI involved in the translational regulation, we constructed mutants (ALD/L/AupSLI and ALD/L/AlowSLI) containing either the upper or the lower region of SLI (Fig. 3A). The deletions on SLI showed no effect on the overall structure of the 3′ UTR predicted with the mfold program. Results showed that the 3′ UTR containing the lower portion of SLI (ALD/L/AlowSLI) had an effect similar to that of wild-type containing the entire SLI (Fig. 3C). The other mutant, with the 3′ UTR containing the upper portion of SLI, (ALD/L/AupSLI) had an activity similar to that of the SLI deletion (ALD/L/AΔSLI) (Fig. 3C).

### The terminal hexamer CGGCCC is the negative regulater in IRES-mediated translation of CSFV

Since the lower portion of SLI contributes to the translation repression, we then generated the mutants containing the left strand sequence (ALD/L/AlowSLI-L) or the right strand sequence (ALD/L/AlowSLI-R) of the lowSLI mutant (Fig. 3A). The RNA transcript containing the left strand of lowSLI (22 nts) resulted in similar translation activity to that of the SLI deletion (Fig. 3D). Although the RNA transcript containing the right strand sequence of lowSLI (17 nts) showed different results, the activity was only 50% that of wild type (Fig. 3D). These results indicate that the primary sequence rather than secondary structure at the very 3′-end of CSFV genome can negatively regulate the translation (Fig. 3D).

**Figure 4:**
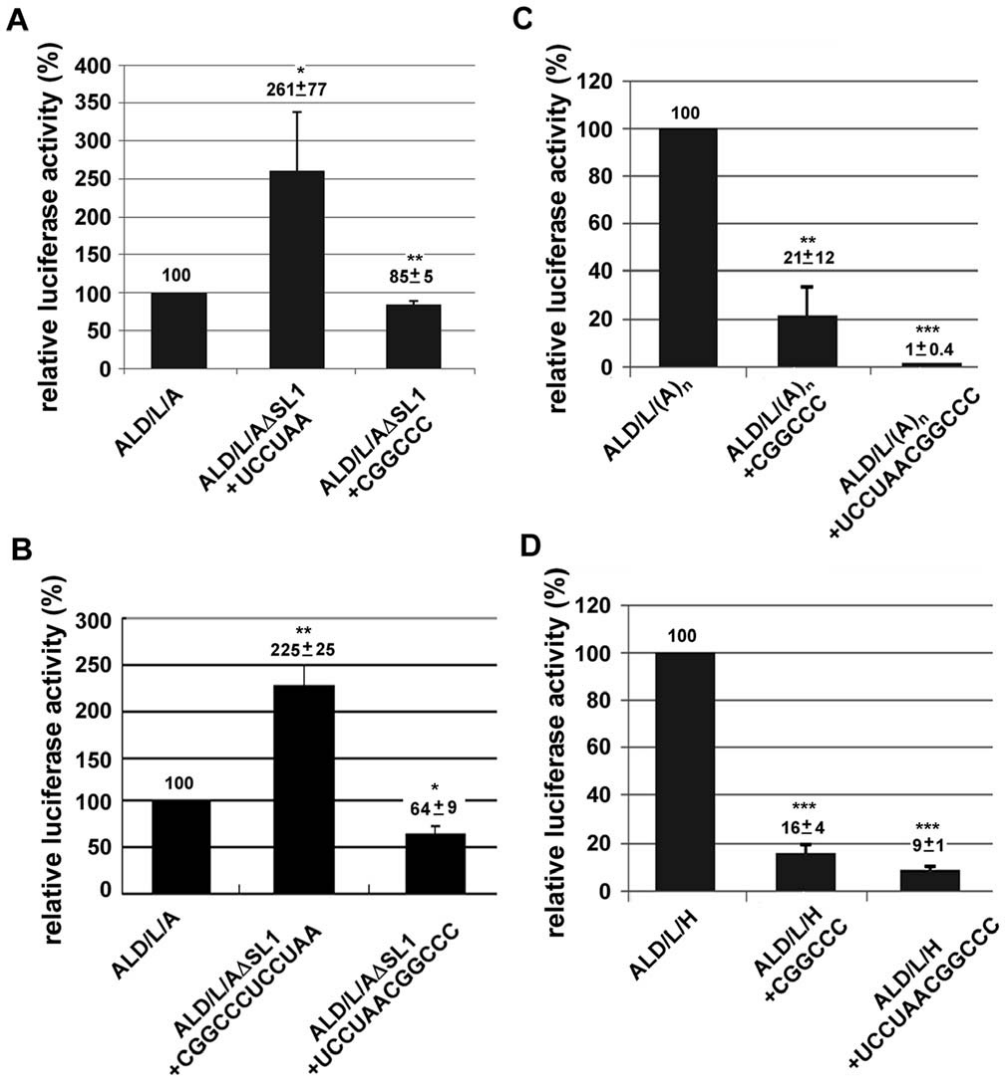
The hexamer sequence at the very 3′-end of the genome is involved in the CSFV IRES-mediated translation. Firefly luciferase activity of the reporter transcripts (1 pmole) containing the wild typ 3′ UTR (ALD/L/A) and its derivatives in **A** and **B**, ALD/L/(A)n and its derivatives in **C**, or ALD/L/H and its derivatives in **D** at 6 hours of post tranfection in PK-15 cells. All the transfection experiments have been independently repeated at least three times. The relative luciferase activity with standard deviations and their significances (*t*-test) was shown above each statistic bar. Asterisks indicate statistically significant differences compared with the control construct indicated as ALD/L/A (*p<0.05, **p<0.01, ***p<0.001).

**Figure 5:**
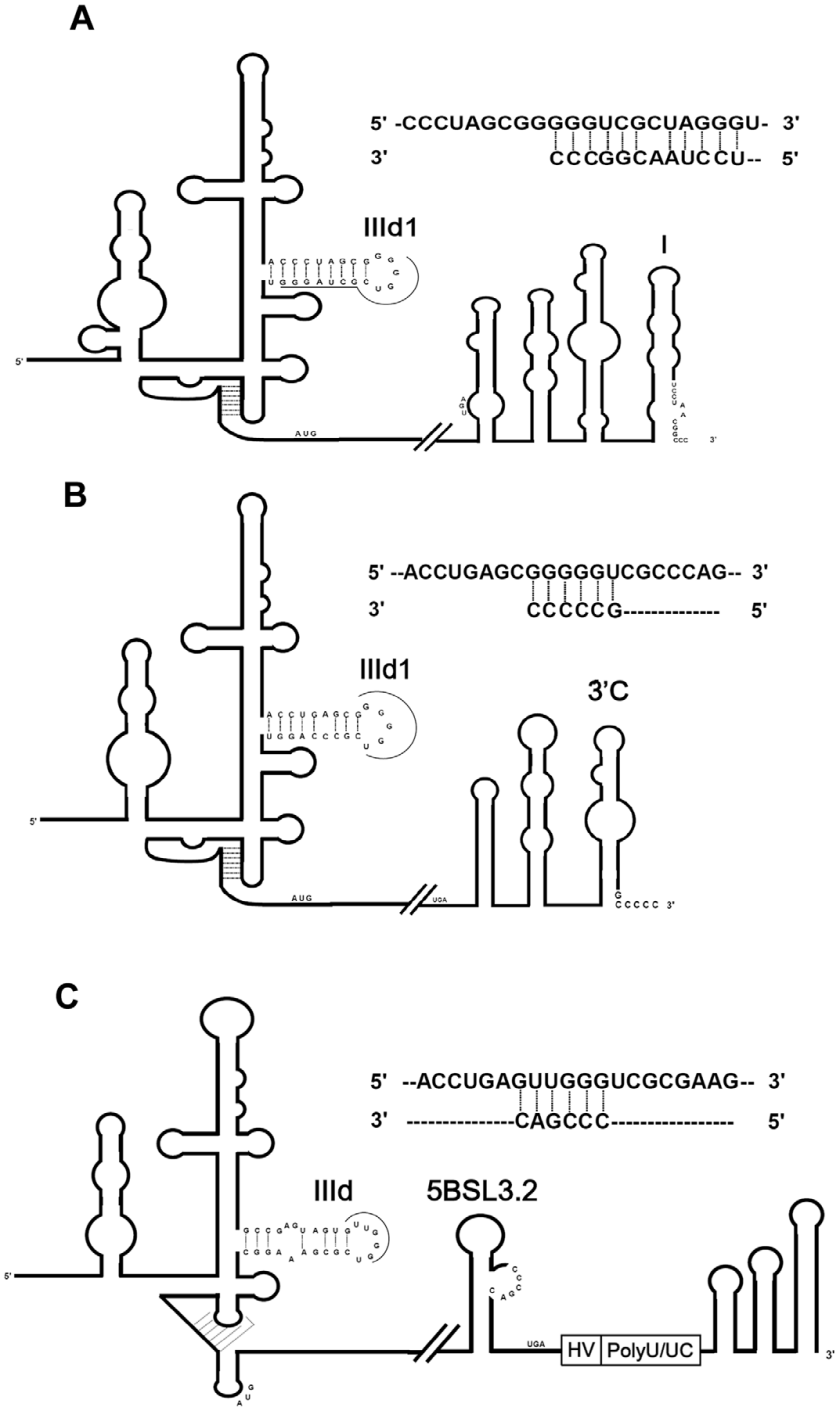
The proposed model for the viral 5′-3′ long distance interaction. The brief diagrams of the genome of CSFV in A, BVDV in B, and HCV in C with the emphasized structures of the 5′ and 3′ untranslated regions were illustrated. The hypothetical bases responding the interaction between the 5′ and the 3′ regions were indicted.

We further dissected this sequence into two parts UCCUAA and CGGCCC to examine their potential effects on translation repression. In the absence of SLI, the RNA harboring CGGCCC hexamer (ALD/L/AΔSLI+CGGCCC) but not UCCUAA (ALD/L/AΔSLI+UCCUAA) responded to the repression (Fig. 4A). We have also tested whether the hexamer CGGCCC should be located in the very 3′-end of the molecture to response in the repression. Results indicate that the hexamer CGGCCC reponding in translation repression is required to be located at the very 3′-end (Fig. 4B). These results also suggested that the single-strand form of UCCUAACGGCCC is more efficient to block the translation than that in the stem-loop form (as in SLI) statistically significant. We have wondered why the very 3′-end sequence influenced the IRES-mediated translation in cells. After a sequence alignment and comparison with the help of the mfold program, we noticed that the very 3′-end sequence 5′UCCUAACGGCCC3′ could have the potential base-pairing to the IIId1 subdomain in the IRES (Fig. 5A). Therefore, we hypothesised that the interaction of the 3′-end sequence to the subdomain IIId1 of IRES in the 5′ UTR downregulated the IRES-mediated translation. The apical loop of sub-domain IIId1 contains the phylogenetically conserved GGG triplet, which is the primary determinant of the 40 S ribosomal subunit binding site in the IRES [38]. Therefore, the 5′-3′ interaction could possibly block the accessibility of the 40 S ribosomal subunit and repress the translation.

We then investigated whether addition of the hexamer to the terminus of the poly(A) tail or the 3′ UTR of HCV could block the CSFV IRES-mediated translation. Our results indicated that the activity of ALD/L/(A)n+CGGCCC and ALD/L/H+CGGCCC was reduced to about 21% and 16% of that of ALD/L/(A)n and ALD/L/H, respectively (Fig. 4C and 4D). When the 3′-ends of ALD/L/(A)n and ALD/L/H were added to the longer sequence UCCUAACGGCCC for base-pairing, the reduction in translation activity was enhanced, down to 1% and 9%, respectively (Fig. 4C and 4D). These results suggest that the 3′-terminal sequence CGGCCC represses the IRES-mediated translation through the 5′-3′ interaction by base-pairing.

## Discussion

In this study, we have demonstrated that the 3′ UTR of CSFV plays a regulatory role in IRES-mediated translation. However, the regulation in CSFV is somewhat different from those of HCV [35], [36], [37], [39] and Dengue virus [40], the other members of the same family. The secondary structure in the 3′ UTR of CSFV can have both up- (SLII and SLIII) and down-regulaing effects (SLI) in translation (Fig. 3). The switch in helping or repressing the translaion may rely on the availability of the 3′-end hexamer sequence CGGCCC. One of the possibilities to shut down the translation is to go through the interaction of the CGGCCC with the IIId1 domain in IRES which has been reported to be the 40 S ribosomal subunit binding site [38]. Mutants ALD/L/(A)n+CGGCCC and ALD/L/H+CGGCCC contain the only sequence (the hexamer) derived from CSFV in their 3′ UTR which could repress the translation by about 80% of that without the terminal hexamer (Fig. 4). We have also observed that the RNA transcripts containing the extra non-viral nucleotides at the very 3′-end (abutting to the CGGCCC) derived from restriction enzyme-linearized plasmids could vary the repression effects when transfected into the cells. The variation effect was overcome when the RNA transcripts with precise ends were all derived from the PCR fragments containing the T7 promoter. The results suggest that the terminal non-viral sequence may interfere with the availability of the CGGCCC sequence. These observations were confirmed by switching the terminal hexamer CGGCCC with its upstream sequence UCCUAA (from 5′-UCCUAACGGCCC3′ to 5′-CGGCCCUCCUAA3′) and losing the repression activity (Fig. 4).

The long-range RNA-RNA interaction of the 5BSL3.2 domain near the 3′-end and the IRES IIId domain located at the 5′ UTR of HCV (Fig. 5C) is involved in modulating the switch from translation to replication [21], [41]. The apical loop of IIId1 in CSFV and BVDV and IIId in HCV containing the phylogenetically conserved GGG triplet (Fig. 5), the primary determinant of the 40 S ribosomal subunits binding site [38], could be blocked by antisense oligonucleotides or RNA aptamer and inhibit the IRES-mediated translation [42], [43]. These results suggested that the apical loop of IRES IIId1 in CSFV and BVDV or IIId in HCV appears to be a control element in regulating the viral gene expression.

Due to the limited size of the viral genome, the viruses have evolved various strategies to modulate their gene expression. It is not surprising that the viral genome harbors the *cis*-regulatory element at the 3′-end of the genome to control the translation apart from the 5′ UTR. When the positive-sense RNA virus infects the target cells, the 3′ UTR can strongly enhance the translation via the 5′-3′ cross-talk, as with those of cellular mRNAs. The cellular proteins such as polypyrimidine tract-binding protein and insulin-like growth factor 2 mRNA-binding protein 1 bind to the 5′- and the 3′-end of HCV RNA that cause the viral RNA cyclization, and therefore facilitate translation [18], [19]. Further, common cellular proteins such as NF45 and RNA helicase A, identified by UV crosslinking assays or RNA-pulldown assays, were found to associate with the 3′ UTRs of CSFV, BVDV, and HCV [18], [20], [44], [45]. These 3′ UTR-binding proteins would be expected to be involved in translation/replication regulation. Since the template for the translation and replication of a positive-sense RNA virus is the same, the switch between these two processes should be in a tight regulation [46]. The availability of the 3′-terminus CGGCCC for the interaction with IRES IIId1 of CSFV in this study could be facilitated by resolving the stem-loop structure in SLI using a cellular protein such as RNA helicase which was reported to interact with the 3′ UTR of CSFV [45]. Finally, it is possible that through the long-range 5′-3′ interaction of CSFV either by base-pairing or with the help of viral [32], [33] or host proteins [45], the translation could be well-regulated.

## Supporting Information

Figure S1 **Enzymatic probing of the 5′ end-labeled LPC 3′UTR SL I.** **A.** Summary of the enzymatic structure probing results of SLI. **B.** The RNAs were treated with RNase A (lane A), RNase T1 (lane T1), RNase T2 (lane T2) and RNase V1 (lane V1). The concentration of enzymes used in each reaction is indicated above each lane. Lane C is the control treatment of the 5′ end-labeled LPC3′UTR with no RNase added and lane Alk is the 5′ end-labeled LPC3′UTR partial digested with alkaline buffer to serve as markers. The cleaved RNA fragments were resolved on a 10% sequencing gel.
(TIF)

Figure S2 **Enzymatic probing of the SLIII region with the 5′ end-labeled LPC 3′UTR.** **A.** Summary of the enzymatic structure probing results of SLIII. **B.** The RNAs were treated with RNase A (lane A), RNase T1 (lane T1), RNase T2 (lane T2) and RNase V1 (lane V1). The concentration of enzymes used in each reaction is indicated above each lane. Lane C is the control treatment of the 5′ end-labeled LPC3′UTR with no RNase added and lane Alk is the 5′ end-labeled LPC3′UTR partial digested with alkaline buffer to serve as markers. The cleaved RNA fragments were resolved on a 10% sequencing gel.
(TIF)

Figure S3 **Enzymatic probing of the SLIV region with the 5′ end-labeled LPC 3′UTR.** **A.** Summary of the enzymatic structure probing results of SLIV. **B.** The RNAs were treated with RNase A (lane A), RNase T1 (lane T1), RNase T2 (lane T2) and RNase V1 (lane V1). The concentration of enzymes used in each reaction is indicated above each lane. Lane C is the control treatment of the 5′ end-labeled LPC3′UTR with no RNase added and lane Alk is the 5′ end-labeled LPC3′UTR partial digested with alkaline buffer to serve as markers. The cleaved RNA fragments were resolved on a 10% sequencing gel.
(TIF)

## References

[1] Moennig V (2000). Introduction to classical swine fever: virus, disease and control policy.. Vet Microbiol.

[2] Paton DJ, Greiser-Wilke I (2003). Classical swine fever–an update.. Res Vet Sci.

[3] Deng MC, Huang CC, Huang TS, Chang CY, Lin YJ (2005). Phylogenetic analysis of classical swine fever virus isolated from Taiwan.. Vet Microbiol.

[4] Pan CH, Jong MH, Huang TS, Liu HF, Lin SY (2005). Phylogenetic analysis of classical swine fever virus in Taiwan.. Arch Virol.

[5] Lin YJ, Chien MS, Deng MC, Huang CC (2007). Complete sequence of a subgroup 3.4 strain of classical swine fever virus from Taiwan.. Virus Genes.

[6] Fraser CS, Doudna JA (2007). Structural and mechanistic insights into hepatitis C viral translation initiation.. Nat Rev Microbiol.

[7] Pestova TV, Shatsky IN, Fletcher SP, Jackson RJ, Hellen CU (1998). A prokaryotic-like mode of cytoplasmic eukaryotic ribosome binding to the initiation codon during internal translation initiation of hepatitis C and classical swine fever virus RNAs.. Genes Dev.

[8] Lukavsky PJ (2009). Structure and function of HCV IRES domains.. Virus Res.

[9] Fraser CS, Hershey JW, Doudna JA (2009). The pathway of hepatitis C virus mRNA recruitment to the human ribosome.. Nat Struct Mol Biol.

[10] Locker N, Easton LE, Lukavsky PJ (2007). HCV and CSFV IRES domain II mediate eIF2 release during 80 S ribosome assembly.. EMBO J.

[11] Kim YK, Back SH, Rho J, Lee SH, Jang SK (2001). La autoantigen enhances translation of BiP mRNA.. Nucleic Acids Res.

[12] Schepens B, Tinton SA, Bruynooghe Y, Beyaert R, Cornelis S (2005). The polypyrimidine tract-binding protein stimulates HIF-1alpha IRES-mediated translation during hypoxia.. Nucleic Acids Res.

[13] Ali N, Siddiqui A (1995). Interaction of polypyrimidine tract-binding protein with the 5′ noncoding region of the hepatitis C virus RNA genome and its functional requirement in internal initiation of translation.. J Virol.

[14] Blyn LB, Towner JS, Semler BL, Ehrenfeld E (1997). Requirement of poly(rC) binding protein 2 for translation of poliovirus RNA.. J Virol.

[15] Costa-Mattioli M, Svitkin Y, Sonenberg N (2004). La autoantigen is necessary for optimal function of the poliovirus and hepatitis C virus internal ribosome entry site in vivo and in vitro.. Mol Cell Biol.

[16] Liu Y, Wimmer E, Paul AV (2009). Cis-acting RNA elements in human and animal plus-strand RNA viruses.. Biochim Biophys Acta-Gene Reg Mech.

[17] Edgil D, Harris E (2006). End-to-end communication in the modulation of translation by mammalian RNA viruses.. Virus Res.

[18] Weinlich S, Huttelmaier S, Schierhorn A, Behrens SE, Ostareck-Lederer A (2009). IGF2BP1 enhances HCV IRES-mediated translation initiation via the 3′UTR.. RNA.

[19] Ito T, Tahara SM, Lai MM (1998). The 3′-untranslated region of hepatitis C virus RNA enhances translation from an internal ribosomal entry site.. J Virol.

[20] Isken O, Grassmann CW, Sarisky RT, Kann M, Zhang S (2003). Members of the NF90/NFAR protein group are involved in the life cycle of a positive-strand RNA virus.. EMBO J.

[21] Friebe P, Boudet J, Simorre JP, Bartenschlager R (2005). Kissing-loop interaction in the 3′ end of the hepatitis C virus genome essential for RNA replication.. J Virol.

[22] Wang L, Jeng KS, Lai MM (2011). Poly(C)-binding protein 2 interacts with sequences required for viral replication in HCV 5′ UTR and directs HCV RNA replication through circularizing the viral genome.. J Virol.

[23] Isken O, Grassmann CW, Yu HY, Behrens SE (2004). Complex signals in the genomic 3′ nontranslated region of bovine viral diarrhea virus coordinate translation and replication of the viral RNA.. RNA.

[24] Silberklang M, Gillum AM, RajBhandary UL (1977). The use of nuclease P1 in sequence analysis of end group labeled RNA.. Nucleic Acids Res.

[25] Zuker M (2003). Mfold web server for nucleic acid folding and hybridization prediction.. Nucleic Acids Res.

[26] Cheng CP, Tsai CH (1999). Structural and functional analysis of the 3′ untranslated region of bamboo mosaic potexvirus genomic RNA.. J Mol Biol.

[27] Thompson JD, Higgins DG, Gibson TJ (1994). CLUSTAL W: improving the sensitivity of progressive multiple sequence alignment through sequence weighting, position-specific gap penalties and weight matrix choice.. Nucleic Acids Res.

[28] Xiao M, Gao J, Wang Y, Wang X, Lu W (2004). Influence of a 12-nt insertion present in the 3′ untranslated region of classical swine fever virus HCLV strain genome on RNA synthesis.. Virus Res.

[29] Bjorklund HV, Stadejek T, Vilcek S, Belak S (1998). Molecular characterization of the 3′ noncoding region of classical swine fever virus vaccine strains.. Virus Genes.

[30] Moormann RJ, van Gennip HG, Miedema GK, Hulst MM, van Rijn PA (1996). Infectious RNA transcribed from an engineered full-length cDNA template of the genome of a pestivirus.. J Virol.

[31] Deng R, Brock KV (1993). 5′ and 3′ untranslated regions of pestivirus genome: primary and secondary structure analyses.. Nucleic Acids Res.

[32] Xiao M, Wang Y, Zhu Z, Yu J, Wan L (2009). Influence of NS5A protein of classical swine fever virus (CSFV) on CSFV internal ribosome entry site-dependent translation.. J Gen Virol.

[33] Zhu Z, Wang Y, Yu J, Wan L, Chen J (2010). Classical swine fever virus NS3 is an IRES-binding protein and increases IRES-dependent translation.. Virus Res.

[34] Bradrick SS, Dobrikova EY, Kaiser C, Shveygert M, Gromeier M (2007). Poly(A)-binding protein is differentially required for translation mediated by viral internal ribosome entry sites.. RNA.

[35] Lourenco S, Costa F, Debarges B, Andrieu T, Cahour A (2008). Hepatitis C virus internal ribosome entry site-mediated translation is stimulated by cis-acting RNA elements and trans-acting viral factors.. FEBS J.

[36] Bung C, Bochkaeva Z, Terenin I, Zinovkin R, Shatsky IN (2010). Influence of the hepatitis C virus 3′-untranslated region on IRES-dependent and cap-dependent translation initiation.. FEBS Lett.

[37] Bradrick SS, Walters RW, Gromeier M (2006). The hepatitis C virus 3′-untranslated region or a poly(A) tract promote efficient translation subsequent to the initiation phase.. Nucleic Acids Res.

[38] Kolupaeva VG, Pestova TV, Hellen CU (2000). Ribosomal binding to the internal ribosomal entry site of classical swine fever virus.. RNA.

[39] Song YT, Friebe P, Tzima E, Junemann C, Bartenschlager R (2006). The hepatitis C virus RNA 3′-untranslated region strongly enhances translation directed by the internal ribosome entry site.. J Virol.

[40] Chiu WW, Kinney RM, Dreher TW (2005). Control of translation by the 5′- and 3′-terminal regions of the dengue virus genome.. J Virol.

[41] Romero-Lopez C, Berzal-Herranz A (2009). A long-range RNA-RNA interaction between the 5′ and 3′ ends of the HCV genome.. RNA.

[42] Tallet-Lopez B, Aldaz-Carroll L, Chabas S, Dausse E, Staedel C (2003). Antisense oligonucleotides targeted to the domain IIId of the hepatitis C virus IRES compete with 40 S ribosomal subunit binding and prevent in vitro translation.. Nucleic Acids Res.

[43] Kikuchi K, Umehara T, Fukuda K, Kuno A, Hasegawa T (2005). A hepatitis C virus (HCV) internal ribosome entry site (IRES) domain III–IV-targeted aptamer inhibits translation by binding to an apical loop of domain IIId.. Nucleic Acids Res.

[44] Isken O, Baroth M, Grassmann CW, Weinlich S, Ostareck DH (2007). Nuclear factors are involved in hepatitis C virus RNA replication.. RNA.

[45] Nadar M, Chan MY, Huang SW, Huang CC, Tseng JT (2011). HuR binding to AU-rich elements present in the 3′ untranslated region of Classical swine fever virus.. Virol J.

[46] Daijogo S, Semler BL (2011). Mechanistic intersections between picornavirus translation and RNA replication.. Adv Virus Res.

