# Supplementary figures and images for: The 3′-Terminal Hexamer Sequence of Classical swine fever virus RNA Plays a Role in Negatively Regulating the IRES-Mediated Translation
Source: PLoS One. 2012 Mar 14;7(3):e33764. doi: 10.1371/journal.pone.0033764 (PMC3303849; doi:10.1371/journal.pone.0033764)

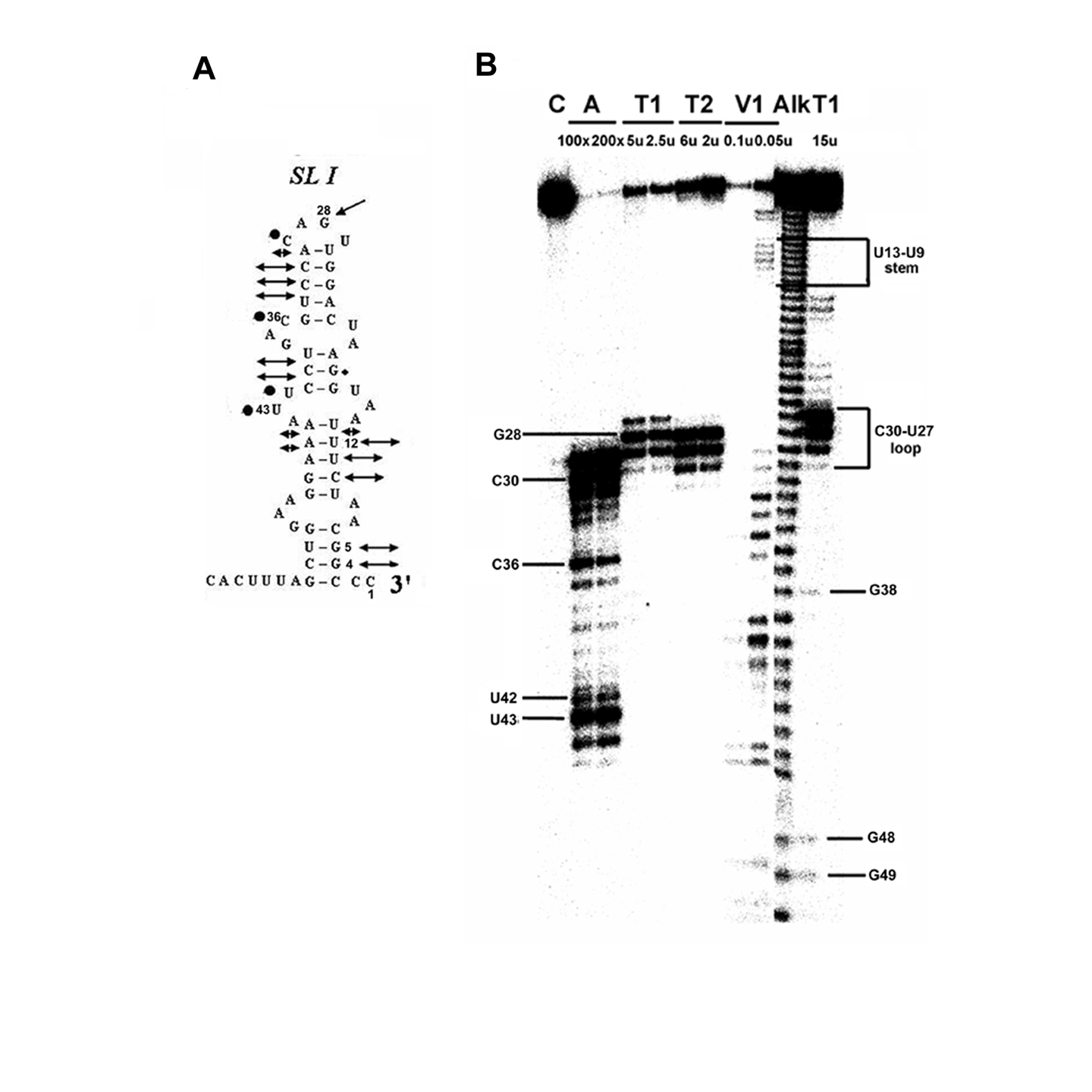

Supplement: Figure S1 — Enzymatic probing of the 5′ end-labeled LPC 3′UTR SL I. A. Summary of the enzymatic structure probing results of SLI. B. The RNAs were treated with RNase A (lane A), RNase T1 (lane T1), RNase T2 (lane T2) and RNase V1 (lane V1). The concentration of enzymes used in each reaction is indicated above each lane. Lane C is the control treatment of the 5′ end-labeled LPC3′UTR with no RNase added and lane Alk is the 5′ end-labeled LPC3′UTR partial digested with alkaline buffer to serve as markers. The cleaved RNA fragments were resolved on a 10% sequencing gel. (TIF) [file pone.0033764.s001.tif]

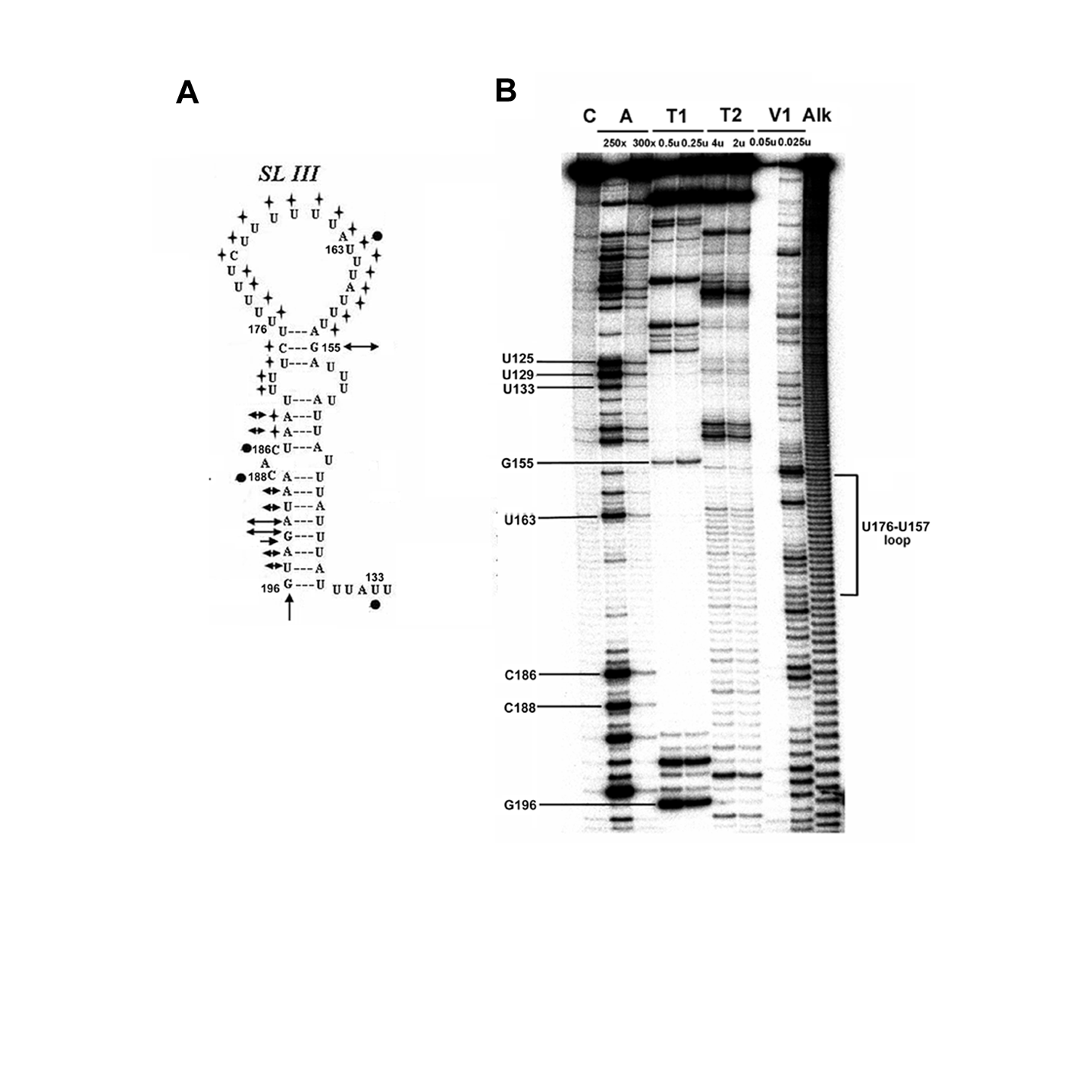

Supplement: Figure S2 — Enzymatic probing of the SLIII region with the 5′ end-labeled LPC 3′UTR. A. Summary of the enzymatic structure probing results of SLIII. B. The RNAs were treated with RNase A (lane A), RNase T1 (lane T1), RNase T2 (lane T2) and RNase V1 (lane V1). The concentration of enzymes used in each reaction is indicated above each lane. Lane C is the control treatment of the 5′ end-labeled LPC3′UTR with no RNase added and lane Alk is the 5′ end-labeled LPC3′UTR partial digested with alkaline buffer to serve as markers. The cleaved RNA fragments were resolved on a 10% sequencing gel. (TIF) [file pone.0033764.s002.tif]

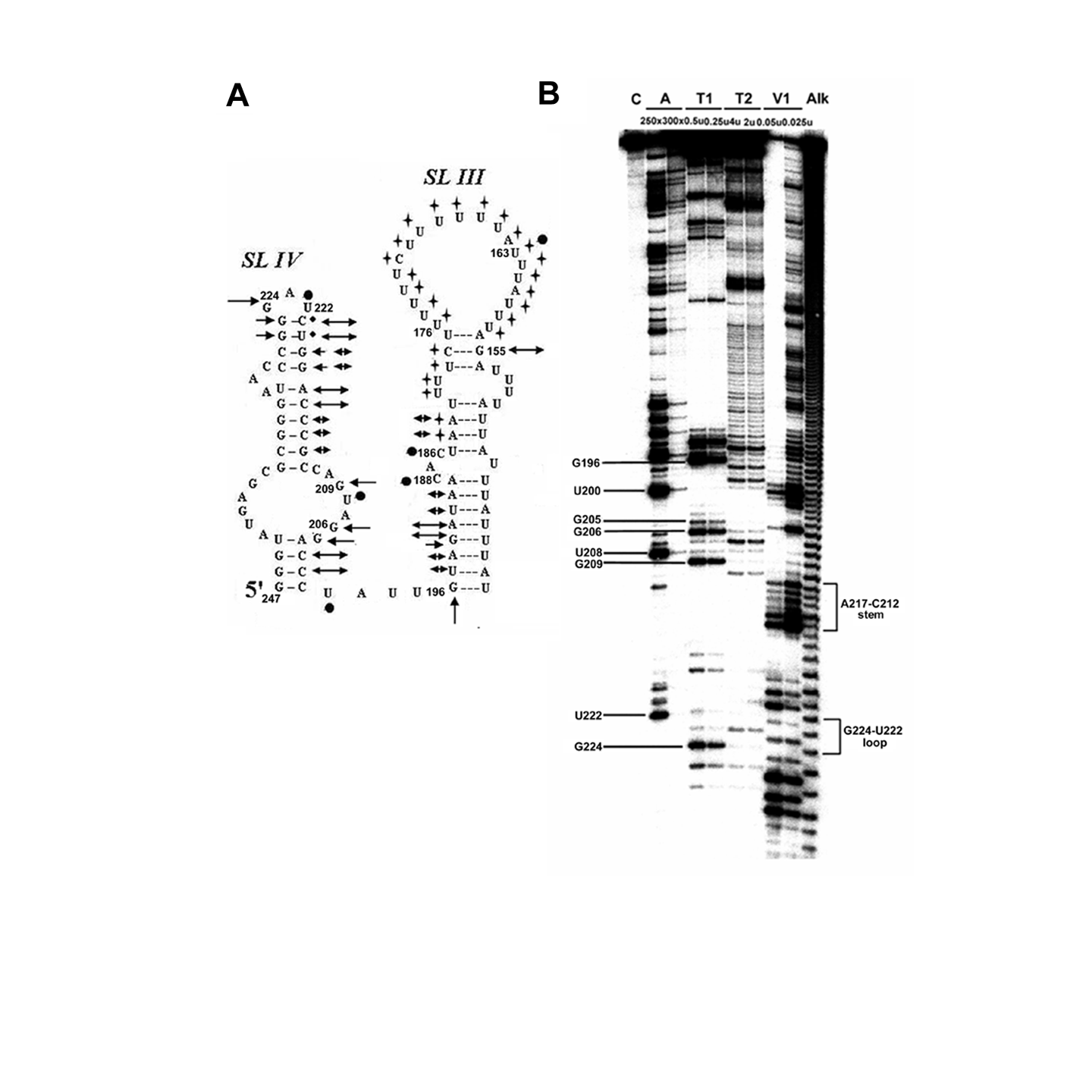

Supplement: Figure S3 — Enzymatic probing of the SLIV region with the 5′ end-labeled LPC 3′UTR. A. Summary of the enzymatic structure probing results of SLIV. B. The RNAs were treated with RNase A (lane A), RNase T1 (lane T1), RNase T2 (lane T2) and RNase V1 (lane V1). The concentration of enzymes used in each reaction is indicated above each lane. Lane C is the control treatment of the 5′ end-labeled LPC3′UTR with no RNase added and lane Alk is the 5′ end-labeled LPC3′UTR partial digested with alkaline buffer to serve as markers. The cleaved RNA fragments were resolved on a 10% sequencing gel. (TIF) [file pone.0033764.s003.tif]
